# Ionising radiation exposure-induced regulation of selected biomarkers and their impact in cancer and treatment

**DOI:** 10.3389/fnume.2024.1469897

**Published:** 2024-10-21

**Authors:** Yonwaba Mzizi, Saidon Mbambara, Boitumelo Moetlhoa, Johncy Mahapane, Sipho Mdanda, Mike Sathekge, Mankgopo Kgatle

**Affiliations:** ^1^ Department of Nuclear Medicine, University of Pretoria and Steve Biko Academic Hospital, Pretoria, South Africa; ^2^ Basic and Translational Research, Nuclear Medicine Research Infrastructure (NuMeRI), Steve Biko Academic Hospital, Pretoria, South Africa; ^3^ Department of Biomedical Sciences, Tropical Diseases Research Centre, Ndola, Zambia; ^4^ School of Health Systems and Public Health, Faculty of Health Sciences, University of Pretoria, Pretoria, South Africa; ^5^ Department of Radiography, University of Pretoria, Pretoria, South Africa; ^6^ Department of Medicine, University of Cape Town and Groote Schuur Hospital, Cape Town, South Africa

**Keywords:** alteration, biomarkers, cancer, genes, ionising radiation, proteins, radiation, treatment resistance

## Abstract

Ionising radiation (IR) is a form of energy that travels as electromagnetic waves or particles. While it is vital in medical and occupational health settings, IR can also damage DNA, leading to mutations, chromosomal aberrations, and transcriptional changes that disrupt the functions of certain cell regulators, genes, and transcription factors. These disruptions can alter functions critical for cancer development, progression, and treatment response. Additionally, IR can affect various cellular proteins and their regulators within different cell signalling pathways, resulting in physiological changes that may promote cancer development, progression, and resistance to treatment. Understanding these impacts is crucial for developing strategies to mitigate the harmful effects of IR exposure and improve cancer treatment outcomes. This review focuses on specific genes and protein biomarkers regulated in response to chronic IR exposure, and how their regulation impacts disease onset, progression, and treatment response.

## Introduction

Ionising radiation (IR) is energy emitted as electromagnetic waves or particles, measured in electron volts (eV) (1). It originates from natural sources like water, soil, and vegetation, as well as artificial sources such as x-rays, gamma rays or particles, and contains higher energy than non-IR (1). Exposure to x-rays and gamma rays is measured in roentgen (R), with one R producing 0.008771 Gray (Gy), defined as the amount of radiation needed to produce ions resulting in a charge of 0.000258 coulombs per kilogram of air under standard conditions (1).

The use of IR is becoming more prevalent in medical and occupational environments. Although it has significantly improved cancer treatment, IR and radiation therapy-prolonged exposure based on absorbed dose and dose rate can damage DNA. This may cause base and sugar damage, as well as single and double strand breaks (SSBs and DSBs). x-rays and gamma rays have low linear energy transfer (LET) and are less densely ionising, while carbon ions have high LET and are more densely ionising. LET levels influence the type of DNA damage, with low LET radiation typically causing about 1,000 SSBs and 40 DSBs. These damages can result in DNA lesions, leading to the loss and rearrangement of genomic sequences. This may alter phenotypic effects, potentially causing malignancy and impacting the effectiveness of cancer treatments (2).

Radiation effects are classified as deterministic or stochastic. Deterministic effects depend on the dose, with higher doses causing more severe outcomes like skin reddening and radiation burns. Stochastic effects are dose-independent and include DNA damage and radiation-induced cancer (3). Diagnostic x-rays, CT scans and radiation therapy can lead to such effects, and potentially leading cancer development, progression and resistance (4). The risk of leukaemia and other secondary cancers increases in adults exposed to radiation from sources like nuclear power plants (5). The first case of radiation-induced cancer was reported in 1902 by Frieben, seven years after the discovery of x-rays (6, 7). Following the Chernobyl disaster in 1986, there were excess cases of cancers, including leukaemia, thyroid cancers, and lymphomas among survivors (8). It is clear that IR causes DNA damage, which can potentially lead to cancer and affect treatment outcomes. Therefore, it is crucial to understand the clinical implications of IR-induced DNA damage and the associated molecular targets to devise more effective preventative and therapeutic strategies. This paper will explore gene and protein biomarkers that are regulated or altered in response to IR-induced DNA damage and their clinical significance in cancer and treatment (also outlined in Table 1).

**Table 1:** Summary of gene and protein-based markers associated with ionising radiation exposure, their functions and clinical relevance.

Gene names	Chromosome	Functions	Clinical significance	Reference
MDM2	12q14 3.q15	DNA responsive gene that serves as a negative regulator and inhibits the tumour suppressing activity of p53 protein.	MDM2 is amplified in 7% of soft tissue tumours, osteosarcomas, and oesophageal carcinomas.	(2, 6)
Flt3	13q12.2	Encodes a tyrosine receptor kinase class 3 that is involved in the regulation of the hematopoesis.	Flt3 is associated with hematopoetic malignancies, and its expression evident in human leukemias.	(9–12)
CDKN1A	6p21.2	Responsible for encoding a cyclin dependent kinase inhibitor and serves as a regulator for the progression of the cell cycle during the G1 phase.	Plays an important role in cell cycle control, DNA damage and apoptosis for cancer progression.	(13, 14)
GADD45	9q22.1-q22.2	Plays an important role in cellular genotoxic and non-genotoxic stress responses acting as stress sensors and tumour suppressors. GADD45 is also important in the induction of apoptosis.	Apparent in the treatment of advanced cervical cancer through external radiation therapy.	(15, 16)
IL-6	7p21	Regulate inflammatory responses which result in immune escape and tumour progression acceleration.	IL-6 may be used as a tumor marker for cancer diagnosis. Elevated levels of IL-6 been associated with advanced stage and metastasis-related morbidity.	(17, 18)
Protein biomarkers				
Protein name	Chromosome	Functions	Clinical significance	
SAA	11p15	Responsible for high density lipoprotein remodelling, lipid metabolism, antibacterial infection, tumour pathology and immune regulation.	High amounts of SAA are associated with metabolic disorders such as diabetes.	(19, 20)
CRP	1q21-q23	Serves as a marker of infection, inflammation, and severe tissue damage.	Elevated levels of CRP indicate systemic inflammatory conditions such as rheumatoid arthritis, lupus or other autoimmune disorders.	(21, 22)
MCP-1	17q12	Responsible in the chemoattraction of blood monocytes and inflammatory process.	MCP-1 is involved in the infiltration of monocytes and macrophages in the TMEs of various types of tumours	(23–25, 26)
APOE	19q13	Responsible for regulating the clearance of lipoproteins from plasma, and lipid transportation to various tissues or cells in the body. APOE isoforms are involved in the modulation of cognitive impairment by radiation exposure.	Elevated levels of APOE increase an individuals’ risk of developing Alzheimer's disease.	(27, 28)
AMY1	1p21.1	Responsible for the secretion of amylase which is required in the digestion of carbohydrates and lipids and is expressed in structures such as submandibular glands, nervous system and pancreas.	AMY1 has been used as a biomarker in the study of ANS dysregulation.	(29–31)
γH2AX	11q23	Responsible for genome stability by signalling DNA damage events and plays an important role in the recruitment and accumulation of DNA repair protein to sites of DSB damage.	Indicated in certain cancers such as breast and endometrial cancer.	(32)
VEGF	6p21.1	VEGF is responsible for encoding a heparin-binding protein that exists as a disulfide linked homodimer.	VEGF is involved in the proliferation and migration of vascular endothelial cells and has been implicated as a driving factor in tumour angiogenesis.	(33–35)

## IR-based DNA damage and repair mechanisms

Stress from internal or external factors can cause DNA damage, including base pair changes, replication errors, and breaks in the DNA double helix. Cells counteract these effects through the DNA damage response (DDR), which signals damage and recruits repair factors (1). DNA repair mechanisms like nonhomologous end-joining (NHEJ) and homologous recombination (HR) are crucial for maintaining genomic stability. NHEJ joins damaged DNA ends with little or no homology, which may cause deletions or insertions, while HR uses the undamaged sister chromatid to accurately repair the DNA.

**Figure 1:**
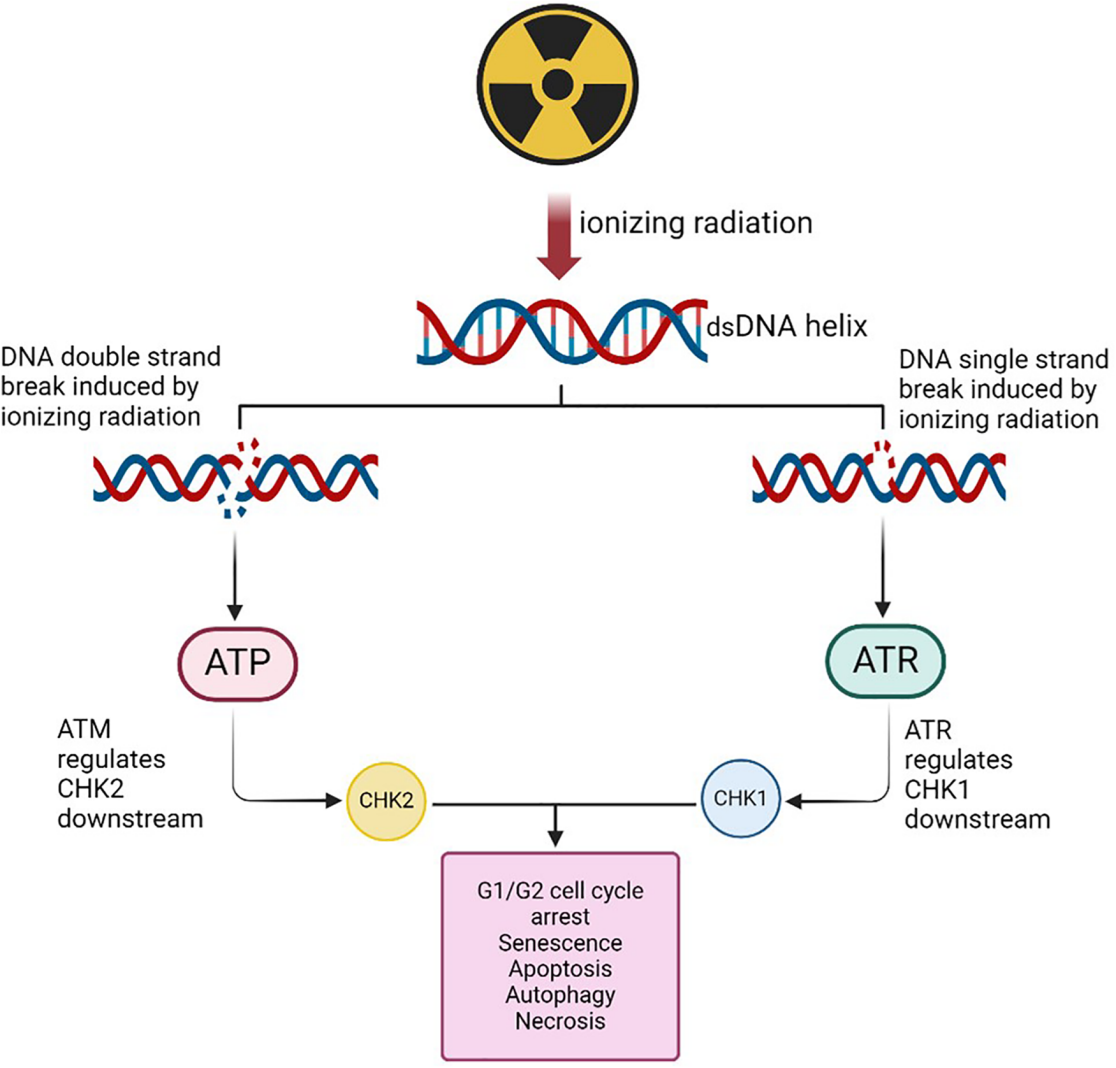
DNA strand breaks due to irradiation may result in the activation of the ATM and the ATR proteins. ATR in turn upregulates the *CHK-1* gene that may be implicated in the cell cycle process in the G1/G2 phase. The ATM is involved in the recruitment of the CHK-2 and affect cell cycle process leading to apoptosis, senescence or autophagy. (Created with BioRender.com, Agreement No: LB27EKK6WL).

The DDR system detects DNA damage using kinases such as ATM and ATR proteins as described in Figure 1. ATM senses double strand breaks (DSBs) caused by IR, while ATR responds to single strand breaks (SSBs) and replication fork stalling due to IR exposure (14, 17, 36).

DNA damage and repair are crucial indicators of the body's response to IR. IR exposure generates reactive oxygen species (ROS), causing damage at specific sites. Repair mechanisms then activate, halting the cell cycle to fix the damage. Successful repair makes cells less radiosensitive, aiding their survival and replication (37). Cells that survive radiotherapy become more resistant to further radiation due to repair mechanisms (38, 39). Radiosensitivity is highest during the G2/M phases and lowest during the late S phase (39, 40). Successful repairs increase resistance to future radiation damage (38). DNA repair involves enzymes like DNA ligase, which mends strand breaks. These processes can lead to genetic changes, potentially contributing to tumorigenesis (41). Epigenetic modifiers, such as DNA methylation and histone modifications, also play a role in regulating DNA repair by affecting nucleosome and chromatin structure (37).

## Gene biomarkers

**Figure 2:**
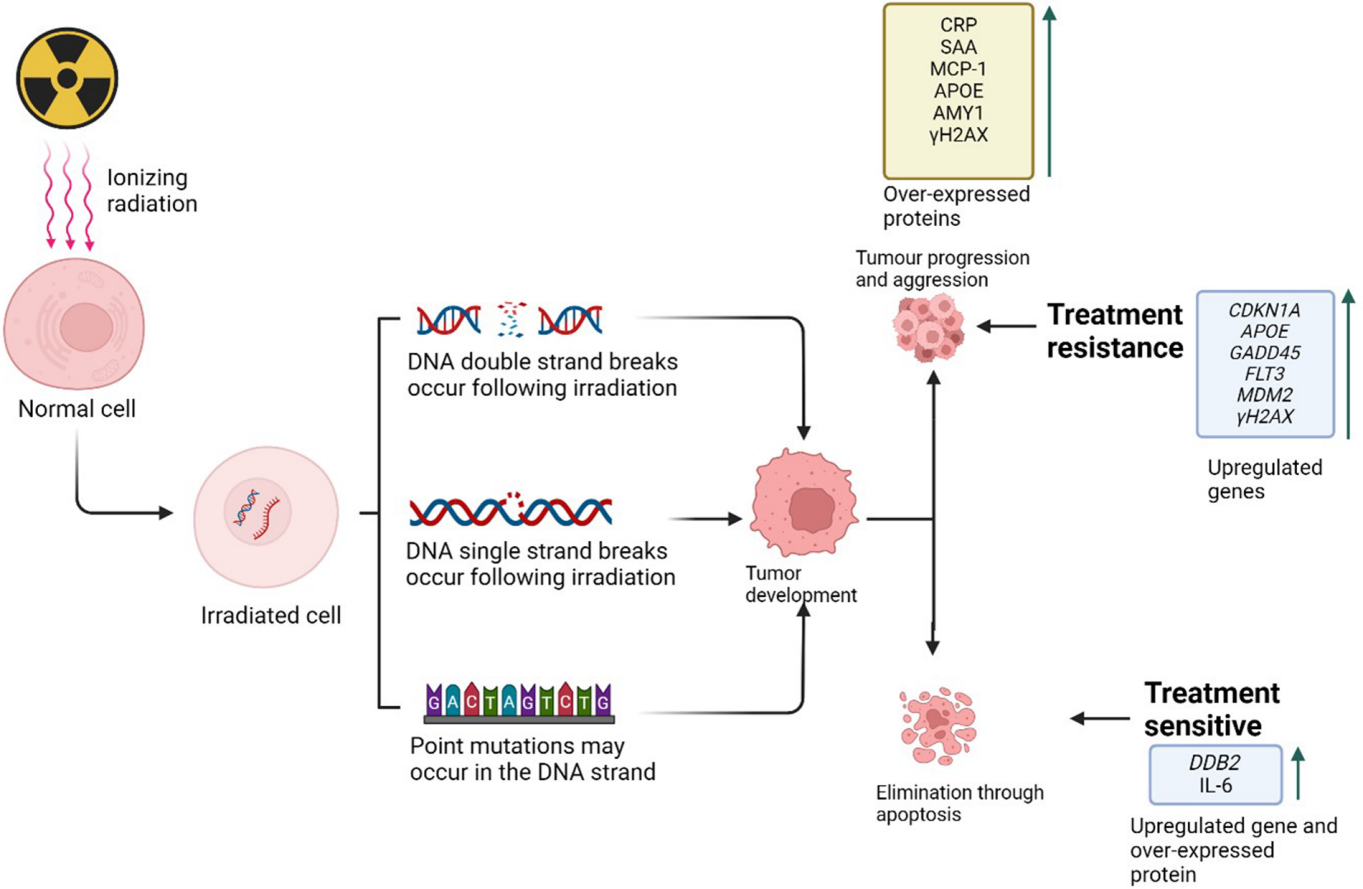
Ionizing radiation-induced DNA damage plays a crucial role in cancer progression and treatment response. This damage includes double-strand breaks (DSBs) and single-strand breaks (SSBs) in irradiated cells, which can lead to the development and progression of cancerous cells. These cells may differentiate and proliferate through the upregulation of various pathways, resulting in either apoptosis or tumor formation, thereby influencing treatment response (Created with BioRender.com, Agreement No: AP27B1MHP8).

IR-induced DNA damage can lead to somatic mutations that disrupt cell regulation and potentially cause cancer, leaving a mutational signature on the cancer cell genome (41). Genes involved in cell cycle regulation, DNA repair, and oxidative stress response are implicated in IR-induced DNA damage, influencing tumour development and response to radiation therapy (41). IR can alter gene expression, with some genes like *murine double minute 2* (*MDM2*), *growth arrest and DNA damage inducible alpha* (*GADD45*), *Flt3 Ligand* (*Flt3l*), and *cyclin dependent kinase inhibitor 1A* (*CDKN1A*) are consistently up regulated, affecting DNA repair and cell cycle control (Figure 2) (41). Long-term exposure can also increase blood biomarkers such as interleukins that are associated with inflammation and cancer risk. Understanding these genes can help develop personalized radiotherapy plans, improving treatment efficacy and minimizing side effects.

## Murine double minute 2

*MDM2* is a gene with four conserved domains that negatively regulates the tumor-suppressing activity of *p53* (6). It is a proto-oncogene amplified in 7% of all cancers, particularly in soft tissue tumours, osteosarcomas, and oesophageal carcinomas. Elevated *MDM2* transcript levels are also found in other cancers without gene amplification (2, 6).

*MDM2* is a transcriptional target of *p53* that regulates E3 ligase activity by binding to, inhibiting, and ubiquitinating *p53* gene, leading to its degradation via the 26S proteasome (2, 4). In the absence of IR, *MDM2* keeps *p53* levels low by preventing its transcriptional activity and tagging it for degradation (2, 4). Upon IR exposure, *p53* is activated, leading to the accumulation and transcription of *p53*-regulated genes through DNA damage response pathways (5).

Elevated *p53* levels and activity in response to ionizing radiation (IR) stimulate *MDM2* overexpression, which helps control cell death (42–44). This was shown in both humanized and non-humanized mouse models irradiated with 4 and 8 Gy for 24 h (45). Another study found increased *p53* levels after 200 rad irradiation in DA-1 murine lymphoma and ML-1 myeloid leukaemia cell lines, leading to *MDM2* release (46). Peak *p53* levels were seen within an hour of radiation exposure, with peak MDM2 levels at 1.5–2 h. A second wave of *p53* was observed hours later, further stimulating *MDM2* expression and counteracting *p53* activities in cells recovering from DNA damage (47). Upregulation of *MDM2* gene was also noted in both *p53*-positive and *p53*-negative mouse tissues before and after γ radiation exposure (46).

In cancer cells with wild type *p53*, a direct correlation between *p53* and *MDM2* levels and the extent of DNA damage from IR was observed (47). A study on cervical cancer treated with radiation therapy found that remaining cancer cells showed contributions from p53 and MDM2, leading to radioresistance (47). Targeting the *p53-MDM2* interaction is seen as a potential cancer treatment strategy. Combining *MDM2* inhibitors with IR could be effective, as supported by a clinical trial using Nutlin-3, which showed increased efficacy and improved outcomes in treating both solid and haematological cancers, particularly enhancing survival in glioblastoma patients when combined with radiation therapy (48).

In experimental mouse models, knocking out *MDM2* gene in embryonic mice was lethal, causing death at 3.5 days post-coitum due to p53-dependent apoptosis, which was prevented by deleting the *p53* gene. Hemizygous MDM2± mice were normal under homeostatic conditions but more sensitive to ionizing radiation compared to wild-type animals (2, 6). This suggests that *MDM2* gene is a crucial regulator of the p53 protein, which controls cell cycle arrest and apoptosis. Without *MDM2 gene*, p53 activity becomes unregulated, leading to excessive apoptosis and embryonic death.

## Flt3 Ligand

Flt3l is a crucial ligand for the Flt3 receptor tyrosine kinase encoded by *Flt3* gene, playing a vital role in blood cell formation by stimulating the growth and differentiation of hematopoietic stem cells (9). It collaborates with other growth factors like G-CSF, GM-CSF, SCF, and IL-3 to develop various blood cell types and promote the proliferation of stem cells and dendritic (10), making it significant in cancer immunotherapy.

The *Flt3* gene encodes the Flt3 ligand, which plays a significant role in response to IR exposure. IR can induce mutations in the *Flt3* gene, particularly internal tandem duplications (ITD) and point mutations (11). These mutations lead to ligand-independent dimerization and constitutive activation of the Flt3 receptor, triggering downstream signalling pathways that promote uncontrolled cell growth (11). This is associated with the development and poor prognosis of acute myeloid leukaemia (AML). Such mutations are found in about 30% of AML patients and a smaller number of patients with acute lymphocytic leukaemia (ALL) or myelodysplastic syndrome (11).

Abnormal plasma *Flt3l* levels indicate radiation exposure-induced damage, making them useful for clinical and emergency assessments (12). A deeper understanding of ionizing radiation-induced damage and *Flt3* gene mutations can aid in developing targeted therapies and improving outcomes for patients with radiation-induced cancers. In cancer therapy, *Flt3l* is being studied to enhance the effectiveness of radiation treatment by boosting the immune response and reducing metastases.

## Growth arrest and DNA damage inducible alpha

*GADD45* is a gene induced by DNA damage and regulated by the p53 protein during the G1 phase of the cell cycle (18, 49). The *GADD45α*, *GADD45β*, and *GADD45γ* genes encoding the GADD45 protein family, are upregulated in response to DNA damage caused by ionizing radiation, playing a significant role in cancer development and treatment (50, 51). For example, lymphoblastoid cells exposed to 3Gy and 10Gy *γ* radiation showed increased *GADD45* gene expression from 1 to 24 h post-exposure (15). When cells are exposed to ionizing radiation, the upregulation of *GADD45* gene aids in DNA repair, cell cycle arrest, and apoptosis, preventing the propagation of damaged cells that could lead to cancer (50, 51).

GADD45 proteins also function as tumour suppressors, maintaining genomic integrity by resolving DNA damage induced by ionizing radiation (52, 53). However, their expression can be altered in response to IR exposure. For instance, a study by Snyder et al. (51) demonstrated that peripheral blood from healthy subjects showed altered expression of various DNA-repair genes, including *GADD45*, following exposure to 0 and 2Gy of x-ray radiation. Similar results were observed in a study by Smirnov et al. (54), where B cells irradiated with 10Gy showed changes in *GADD45* expression at 2- and 6-h post-irradiation. Reduced expression of *GADD45* tumour suppressors is associated with genomic instability and increased mutation rates, potentially leading to cancer progression.

Aberrant expression of *GADD45* tumour suppressors can also affect how cancer cells respond to radiation therapy (55). For instance, upregulation of *GADD45A* has been shown to enhance the effectiveness of radiotherapy by increasing the sensitivity of human tongue squamous carcinoma cell lines to IR. Conversely, inactivation of *GADD45A* can make cancer cells more resistant to radiation, impacting treatment outcomes. Overexpression of *GADD45* gene has been linked to increased lethality in cervical cancer in response to radiation therapy. This finding is supported by Asuthkar et al. (16), who demonstrated that *GADD45* induction by IR sensitises medulloblastoma cells to radiation treatment. Understanding the role of *GADD45* can help develop better therapeutic strategies to improve cancer treatment outcomes.

## Cyclin dependent kinase inhibitor 1A

The *CDKN1A* gene is responsible for encoding a cyclin dependent kinase inhibitor and serves as a regulator for the progression of the cell cycle during the G1 phase. The expression of *CDKN1A* gene is governed by the p53 protein and plays a critical role in the cellular response to DNA damage, including damage caused by IR. P53 becomes activated in response to ionizing radiation exposure. This leads to overexpression of C*DKN1A* that triggers cell cycle arrest, particularly at the G1 phase, preventing cells with damaged DNA from proliferating. If the damage has not been resolved or repaired, *CDKN1A* gene promotes apoptosis and cellular senescence to eliminate damaged cells that could potentially cause cancer (56). The transcription of the *CDKN1A* gene reaches its peak 4 h after radiation exposure, while the *DDB2* gene*,* also crucial for DNA damage repair, peaks at 24 h post-irradiation (18).

*DDB2* works in synergy with *CDKN1A*, and usually becomes overexpressed following *DDB2* in response to IR induced DNA damage to enhance nucleotide excision repair pathway to initiate the repair of DNA lesions (57). Elevated levels of CDKN1 and functional p53 are linked to increased cell cycle arrest and apoptosis, which help eliminate cancer cells and result in greater sensitivity to radiation therapy. On the other hand, upregulation in *DDB2* gene may facilitate the repair of radiation-induced DNA damage, leading to resistance in treatment response and aggressiveness in melanoma cancer (58). Amundson et al. (41) demonstrated that *CDKN1A* mRNA levels, along with mRNA for *discoidin domain receptor tyrosine kinase 2* (*DDR2*), *xeroderma pigmentosum C* (*XPC*), *tumour necrosis factor- related apoptosis-inducing ligand* (*TRAIL receptor 2*), *four and a half LIM Domain protein 2* (*FHL2*), *cyclin G* and other cyclin proteins, peak between 12 and 24 h post-irradiation (59–62). This suggests that CDKN1A may work in conjunction with other cell cycle regulators in response to IR. Understanding the interactions among these genes can help develop more effective therapeutic strategies.

Checkpoint proteins such as CHK1 and CHK2 are crucial for the cellular response to IR, impacting DNA repair, cell cycle regulation, and cancer treatment outcomes. They transmit signals from ATM and ATR to facilitate DNA repair (Figure 2). CHK1 is typically active during the synthesis (S) and G2 phases of the cell cycle in response to DNA damage caused by IR (63). CHK1 and CHK2 contribute to the intrinsic resistance mechanisms against radiotherapies that damage DNA. Inhibiting their activities can help develop more effective therapeutic strategies by sensitizing tumours to radiation and preventing the repair of radiation-induced DNA damage.

## Protein biomarkers

Proteins, which are polymers of amino acids linked by peptide bonds, can serve as biomarkers indicating DNA damage from environmental factors like IR. These protein biomarkers operate at the post-translational level, involving modifications that occur after protein synthesis. Such post-translational modifications (PTMs) can greatly influence protein function, localisation, stability, and interactions. Proteins can become overexpressed in response to IR, influencing oncogenes and tumor suppressors, and affecting cell growth and survival pathways. These proteins can serve as biomarkers for predicting cancer development, progression, and treatment sensitivity or resistance Figure 2. Identifying and understanding these biomarkers in the context of IR and cancer can enhance our knowledge of how IR impacts cancer and aid in predicting radiosensitivity, optimizing radiation therapy, and improving treatment outcomes.

## C-reactive protein

C-reactive protein (CRP) is a protein marker produced by the liver in response to inflammation and severe tissue damage (64). CRP levels increase with both the dose and duration of radiation exposure (64). For instance, blood CRP levels rose in 30 rhesus monkeys exposed to 1–8.5 Gy of gamma cobalt-60 radiation (19). A borderline significant increase in CRP levels was observed in radiological technologists who had undergone prior radiation therapy, depending on the dose (63). Similarly, CRP levels in peripheral blood mononuclear cells of mice increased progressively with the dose and time after total body irradiation with 1–7 Gy of gamma radiation (64).

Studies have shown that radiotherapy, which involves high doses of IR, is associated with increased CRP levels and a higher risk of inflammatory diseases (28). Radiation pneumonitis, an inflammatory clinical outcome and a dose-limiting toxicity of radiation therapy, is a side effect of both chemotherapy and radiation therapy. In a separate study, elevated CRP levels were detected in association with inflammatory conditions, including autoimmune diseases (such as hypothyroidism, hyperthyroidism, rheumatoid arthritis, type-1 diabetes, and Crohn's disease) and non-autoimmune diseases (such as type-2 diabetes and osteoarthritis), suggesting that CRP is a marker of the risk of developing an inflammatory condition following radiation therapy (28).

Research has also demonstrated that elevated CRP levels are observed in response to inflammation resulting from IR exposure. This suggests an activation of inflammatory responses and chronic inflammation that could lead to carcinogenesis (65). For instance, a strong correlation between abnormal CRP levels and IR was reported in both breast and prostate cancers (21, 22). Furthermore, high CRP levels have been linked to increased risks of breast, lung, and colorectal cancers (66).

CRP contributes to cancer development by fostering an inflammatory environment, promoting tumour growth and survival, and modulating immune responses. Elevated CRP levels can indicate cancer risk and progression. Chronic inflammation from high CRP levels supports tumour growth, angiogenesis, and metastasis (66, 67). CRP also enhances tumour cell proliferation and survival by protecting them from therapy-induced apoptosis (67). In its monomeric form (mCRP), CRP activates inflammatory mechanisms by interacting with cell membranes and immune cells, aiding initial defence against tumours and creating a carcinogenic environment (68). High baseline CRP levels in healthy individuals are linked to increased future cancer risk, making CRP a potential biomarker for cancer risk and progression (69).

## Monocyte chemotactic protein 1

Monocyte chemotactic protein 1 (MCP-1), is a chemokine essential for the immune system, recruiting monocytes to areas of damage, inflammation, and tumours (23). When the body is exposed to ionizing radiation, MCP-1 is activated to help repair the damage by attracting immune cells to the affected site, contributing to a pro-inflammatory environment that supports tumour growth, progression, and metastasis (23, 24). MCP-1 initiates a proinflammatory response by activating signalling pathways like NF-κB, which are involved in inflammation and can drive tumour progression (25). It has a dual role, capable of either promoting or inhibiting tumour growth depending on the tumour microenvironment. While MCP-1 can support tumour progression by recruiting TAMs and encouraging angiogenesis, it can also boost anti-tumour immune responses under certain conditions (23).

Production of MCP-1 can also be induced in response to radiation therapy for cancer treatment, potentially leading to an inflammatory environment that supports tumour survival and resistance to therapy (23). For example, radiation-induced MCP-1 has been shown to correlate with lung toxicity and inflammation, complicating treatment outcomes in patients with non-small cell lung cancer (NSCLC) (70). Additionally, MCP-1 has been implicated in breast cancer metastasis, particularly to the lungs and brain (26).

To mitigate the effects of MCP-1 during radiation therapy, several strategies can be employed. Anti-inflammatory drugs like corticosteroids can reduce inflammation and MCP-1 levels, minimising the pro-inflammatory environment that supports tumour growth. Specific chemokine inhibitors targeting MCP-1 or its receptor, CCR2, can block its activity and reduce immune cell recruitment to the tumour site (71). Radiation techniques such as fractionated radiation therapy (delivering smaller, frequent doses) and targeted techniques like stereotactic radiosurgery (SRS) and intensity-modulated radiation therapy (IMRT) can minimise MCP-1 production and spare healthy tissue (72). Combining radiation with immunotherapy or chemotherapy agents that have anti-inflammatory properties can enhance anti-tumour responses and mitigate MCP-1's effects. Additionally, maintaining a balanced diet rich in anti-inflammatory foods and engaging in regular, moderate exercise can help manage inflammation and improve overall immune function during radiation therapy.

## Serum amyloid A

Serum amyloid A (SAA) is a vital biomarker for both x-ray and *γ*-ray radiation exposure, as well as cancer progression. Its levels can reflect the extent of radiation-induced inflammation and provide insights into cancer prognosis and treatment responses. SAA levels in the blood can significantly increase in response to IR, such as x-rays and γ-rays (73). As part of the body's acute phase response to IR damage, SAA triggers inflammation. Studies have shown that SAA levels can rise 10–100 times shortly after IR exposure, with levels remaining elevated depending on the severity of inflammation (61). In a study by Huang et al. (20), the expression of SAA during early and late radiation-induced inflammation was assessed in mice exposed to various doses of radiation (1, 2, 4, 8, and 12 Gy) at different time points. It was found that SAA levels moderately increased at 6 h post-irradiation, peaked at 12 h across all doses, and further increased between days 5 and 7 (20). Additionally, significant increases in SAA levels were observed 24 h after total body irradiation with 1–8 Gy of x-rays. These findings suggest that SAA levels increase in a dose-dependent manner following radiation exposure, indicating its potential as a biomarker for assessing radiation exposure and the resulting inflammatory response (73).

SAA plays roles in high-density lipoprotein remodelling, lipid metabolism, antibacterial infection, tumour pathology, and immune regulation (20). Elevated SAA levels often correlate with poor clinical outcomes in various cancers, including renal cell, lung, breast, ovarian, and prostate cancers (19, 74). Aberrant SAA levels drive inflammation, leading to cell proliferation, cancer progression, angiogenesis, and metastasis. While the clinical significance of radiation-induced protein markers in cancer is generally limited, SAA is gaining recognition in the context of lung cancer treatment with radiation therapy. In lung cancer, high SAA levels have been identified as a predictive biomarker for patients at risk of developing radiation pneumonitis following radiation therapy (27).

## Interleukins

Interleukin (IL) proteins are part of the cytokine superfamily, consisting of 38 different types of ILs, and they facilitate interactions between cells (17). These cytokines interact with various elements, including cancer stem cells, epithelial-mesenchymal transition (EMT), and miRNAs, during tumorigenesis. Among them, IL-6 is the most well-recognized for its role in cellular functions. IL-6 is a multifunctional, pleiotropic cytokine in the IL-6 family, involved in the growth and differentiation of B and T lymphocytes. It has been shown to transform human mammospheres and pre-malignant mammary epithelial cells *in vitro*, making them tumorigenic *in vivo (*17).

In the context of IR, IL-6 has been implicated in cancer progression and resistance to treatment by reducing oxidative stress and DNA damage (75). Breummer et al. (18) reported a significant increase in IL-6 following radiation exposure of myeloid cells at doses below 20 Gy. Upregulation of IL-6 enhances the mobility and tumorigenesis of breast cancer epithelial cells in response to IR. In mouse mammary glands, IL-6 is frequently expressed after IR exposure and is produced by IR-senescent fibroblasts. Additionally, epithelial cells, primary human mammospheres, and pre-malignant mammary epithelial cell lines show increased IL-6 expression following IR exposure. Elevated IL-6 levels are linked to the radiation response in prostate cancer, glioblastoma, liver cancer, and lung cancer, highlighting its significant role in cancer therapy (76, 77).

Other serum-based protein markers may include interleukin-22, insulin-like growth factor binding protein-1 (IGFBP-1), IGFBP-3, insulin-like growth factor 1 (IGF-1) and leukaemia inhibitor factor (LIF) (32). For instance, Wei et al. (32) demonstrated an aberrant accumulation of *interleukin-22*, *IGFBP-1*, *IGFBP-3, IGF-1* and *LIF* in the sera of 2-month-old mice irradiated through exposure with carbon atoms which contain an energy of 80 MeV at a rate of 0.25 Gy per min (32), indicating their potential roles as biomarkers in IR exposure.

## Salivary Alpha Amylase

Salivary Alpha Amylase (AMY1) is crucial in the context of radiation exposure, with abnormal levels linked to cancer and its treatment. Stress typically activates the sympathetic nervous system, releasing stress hormones like adrenaline and cortisol, which in turn stimulate the production and release of AMY1 in saliva (29).

AMY1 responds to stress in different ways depending on the stress level. During acute stress, an immediate “fight-or-flight” response occurs, leading to a rapid increase in AMY1 levels in saliva as part of the first line of immune defence (29). Conversely, prolonged or chronic stress, which affects digestion and immune function, results in sustained elevated levels of AMY1 (29).

Recognised as a stress marker, AMY1 levels can become irregular due to radiation therapy. For instance, radiation therapy for head and neck cancer has been shown to alter AMY1 levels and salivary gland function. Enzyme activity assays in patients undergoing radiation therapy for neck and head cancer demonstrated that radiation-induced alterations in AMY1 lead to changes in saliva production (30). Elevated levels of circulating AMY1 have been linked to a higher risk of lung, breast, ovarian, and gastric cancers, significantly associated with increased cell proliferation and metastasis (31). The simultaneous activation of the phosphatidylinositol 3-kinase (PI3 K)/protein kinase B (AKT) and mitogen-activated protein kinases (MAPK) signalling pathways by AMY1 fosters a robust environment for tumour growth, resistance to apoptosis, and metastasis (78, 79). In certain cancers, AMY1 is thought to have antiproliferative effects that inhibit tumour cell growth by regulating various pathways. It can block signalling pathways that promote cancer cell proliferation, thereby slowing or halting their growth and inducing apoptosis (79). AMY1 also modifies the tumour microenvironment, making it less favourable for cancer cell survival by altering cytokine and growth factor levels (79). Furthermore, AMY1 activates pathways such as PI3K/Akt and MAPK, which help suppress tumour growth and metastasis (79). Therefore, AMY1 could serve as a potential biomarker for evaluating the impact of radiation therapy on salivary glands and overall stress levels in patients. Understanding AMY1's role in cancer and radiation exposure might lead to new therapeutic strategies to alleviate side effects and improve patient outcomes.

## Murine double minute 2

At the post-translational level, MDM2 also serves as a crucial protein biomarker involved in the cellular response to IR exposure, potentially contributing to cancer development and metastasis. When cells are exposed to IR, MDM2 expression increases in a p53-dependent manner, helping manage the cell's response to DNA damage. MDM2 promotes cell survival by limiting p53's apoptotic function during IR exposure.

As the primary negative regulator of the p53 tumour suppressor protein, MDM2 inhibits p53's activity by promoting its ubiquitination and degradation. This inhibition prevents p53 from inducing cell cycle arrest and apoptosis in response to IR, leading to tumour formation through the proliferation of damaged cells. Inhibiting MDM2 can enhance the sensitivity of cancer cells to radiation, improving the effectiveness of radiotherapy.

Additionally, MDM2 acts as an oncogene, with its overexpression driving cancer progression by promoting cell proliferation and survival. It also contributes to metastasis and tumour growth by enhancing the invasive capabilities of cancer cells and their spread to other parts of the body.

Furthermore, MDM2 can alter the tumour immune microenvironment, helping cancer cells evade immune detection and destruction, complicating the immune system's ability to combat cancer. High levels of MDM2 are linked to resistance to therapies such as chemotherapy and immunotherapy. Targeting MDM2 with specific inhibitors is being explored to overcome this resistance and improve treatment outcomes.

## Apolipoprotein-E

The apolipoprotein-E (APOE) protein, encoded by the APOE gene, is crucial for lipid metabolism, regulating the clearance of lipoproteins from plasma and transporting lipids to various tissues and cells (64). APOE also plays a role in the body's response to IR, managing the inflammatory response and aiding in the repair and recovery of neural injuries in the central nervous system (80–83). Studies have shown that APOE can influence behavioural impairment following radiation exposure (82, 83). For example, a study by Higuchi et al. (82) found that both APOE knockout and wild-type mice experienced impaired motor coordination and stamina after receiving 2 Gy of total body irradiation. While these effects resolved in wild-type mice by 60 days post-irradiation, they persisted in knockout mice (82). Additionally, knockout mice showed reduced exploratory activity up to 186 days post-treatment, unlike wild-type mice. These findings highlight the role of APOE in the recovery and repair of radiation-induced injury in the central nervous system (82).

Overexpression of APOE has been observed in brain regions like the prefrontal cortex, amygdala, and hippocampus of rhesus macaques in response to radiation therapy, suggesting a role in the brain's response to radiation (83). APOE expression varies among tumor types and cancer cell lines, and it has been shown to have prognostic value and influence treatment outcomes in cancers such as lower-grade glioma, kidney renal clear cell carcinoma, and kidney renal papillary cell carcinoma (84).

## Phosphorylated H2A Histone Family Member X

Phosphorylated H2A Histone Family Member X (γH2AX) is a modified protein, and a recognized marker of DNA double-strand break (DSB) damage caused by radiation, playing a crucial role in cancer research and treatment (21). Indirect ionisation of oxide species generates free radicals, often leading to cell death from IR exposure by breaking down the DNA backbone and creating DSBs. These breaks result in the phosphorylation of histone H2A (H2AX) on serine 139, forming γH2AX, an early indicator of DNA damage post-IR exposure (21).

γH2AX acts as a docking site for various DNA repair proteins at DSB sites in cells exposed to IR. Proteins such as MDC1, 53BP1, and BRCA1 are crucial for making DNA DSBs accessible to the repair machinery. The formation of IR-induced γH2AX activates ATM, ATR, and DNA-PK, which phosphorylate H2AX to enhance the DNA damage signal and coordinate the repair process (21, 22, 85). In this context, ATM is the primary mediator, activated by autophosphorylation at Serine 1981. ATR phosphorylates H2AX during SSBs and DNA replication, while DNA-PK does so during DNA fragmentation and under hypertonic conditions (86). Additionally, the DNA damage response mechanisms are triggered, leading to cell cycle arrest, allowing the cell to repair the damage, survive, and maintain genomic integrity before division.

Abnormal levels of γH2AX may be linked to genomic instability, potentially leading to genetic mutations implicated in cancer development, progression, and treatment outcomes. Human skin cells exposed to radiation (4 Gy of 6 MeV electrons) showed elevated γH2AX levels and increased 53BP1 foci, indicating DNA damage. This damage, including single and double-strand breaks, was observed in various skin cell types and persisted for weeks, leading to fibrosis, a hallmark of cancer development. Additionally, higher γH2AX levels were noted in triple-negative breast cancer cell lines compared to non-triple-negative ones when irradiated with 10 Gy, with γH2AX present in all 54 breast cancer cell lines tested (87, 88). Mice exposed to gamma radiation for 24 h showed elevated γH2AX levels, strongly correlating with unrepaired DNA DSBs observed via γH2AX foci and radiogenic lung cancer. While increased γH2AX may act as a tumour suppressor and promote cell cycle arrest and senescence in premalignant lesions, it is often associated with poor prognosis and tumour aggressiveness (33). Nonetheless, γH2AX is a valuable biomarker for detecting and assessing IR-induced DNA damage and response to cancer radiation therapy.

## Vascular endothelial growth factor (VEGF)

VEGF is a vital protein that promotes the formation of new blood vessels, especially in response to IR to repair damaged vessels. IR generates oxidative stress by producing reactive oxygen species (ROS) and free radicals, which activate signalling pathways such as the epidermal growth factor receptor (EGFR). This activation triggers downstream pathways, including MAPK, resulting in increased VEGF production.

Although VEGF itself does not directly cause cancer, its induction by IR can contribute to the progression and aggressiveness of existing tumours by providing them with essential nutrients and oxygen (34). For instance, radiation-induced hypoxia, caused by damaged blood vessels within tumours, can lead to increased VEGF production through the activation of hypoxia-inducible factor 1-alpha (HIF-1α) (35). HIF-1α is a transcription factor that stabilizes and activates under hypoxic conditions, promoting VEGF production to form new blood vessels necessary for supplying the tumour with oxygen and nutrients (35). VEGF can also bind to VEGF receptors 1 and 2 on the surface of newly formed blood vessels, activating several additional signalling pathways that stimulate cell proliferation, migration, and survival. This process can potentially lead to tumour progression and treatment resistance by allowing new blood vessels to repair radiation-induced damage and support tumour regrowth (89).

However, pharmacologically blocking VEGF has been shown to improve patient outcomes by reducing angiogenesis and enhancing tumour oxygenation. This involves using anti-VEGF agents like bevacizumab in combination with radiation therapies. This approach reduces VEGF activity, leading to decreased angiogenesis and improved effectiveness of radiation therapy against tumour cells (90).

In summary, VEGF plays a dual role in cancer treatment with IR. It promotes blood vessel formation and can lead to tumour resistance. However, using anti-VEGF therapies can enhance radiation treatment effectiveness and improve cancer control.

## Limitations

Identifying biomarkers for radiation exposure that are applicable across various scenarios and types of radiation is challenging. Despite numerous studies over the years, no biomarkers have been specifically validated for radiation exposure alone. Additionally, the lack of data from past nuclear attacks or accidents, which needs to be collected over a specific period following radiation exposure, has significantly hindered progress in researching potential biomarkers. Most studies to date have focused on the effects of whole-body radiation exposure, with very few examining the irradiation of specific body parts. Therefore, the effects of partial body exposure also need to be investigated. Extensive and innovative research is required to develop effective biomarkers for radiation exposure.

## Conclusion and future research opportunities

IR seems to influence various genes and transcriptional regulators that control the cell cycle in ways that are linked to cancer development, progression and resistance to treatment. While there are safety measures in place to manage occupational IR exposure, it is crucial to understand the genomic effects that may contribute to diseases, particularly cancer and treatment response. This understanding will help ensure that additional safety and therapeutic measures can be implemented if needed.

Assessing biomarkers after IR exposure has significant potential for cancer treatment and prognosis. These biomarkers can help design compounds that enhance radiosensitivity, support DNA repair, and provide radioprotection. They can also evaluate the risk of cancer development in organs and tissues at different IR doses. Advanced technologies like next-generation sequencing are needed to identify new biomarkers on a genome-wide scale, aiding in cancer prevention and therapy. Understanding IR-induced organ and tissue injury is crucial for managing cancer patients, and developing models to measure IR exposure is essential for monitoring its long-term effects in clinical research.
